# Supplementary material for: Treatment of Mouse Infants with Amoxicillin, but Not the Human Milk-Derived Antimicrobial HAMLET, Impairs Lung Th17 Responses
Source: Antibiotics (Basel). 2023 Feb 20;12(2):423. doi: 10.3390/antibiotics12020423 (PMC9952748; doi:10.3390/antibiotics12020423)
Supplement: Supplementary file 1 [file antibiotics-12-00423-s001.zip › antibiotics-2195183-supplementary.pdf]

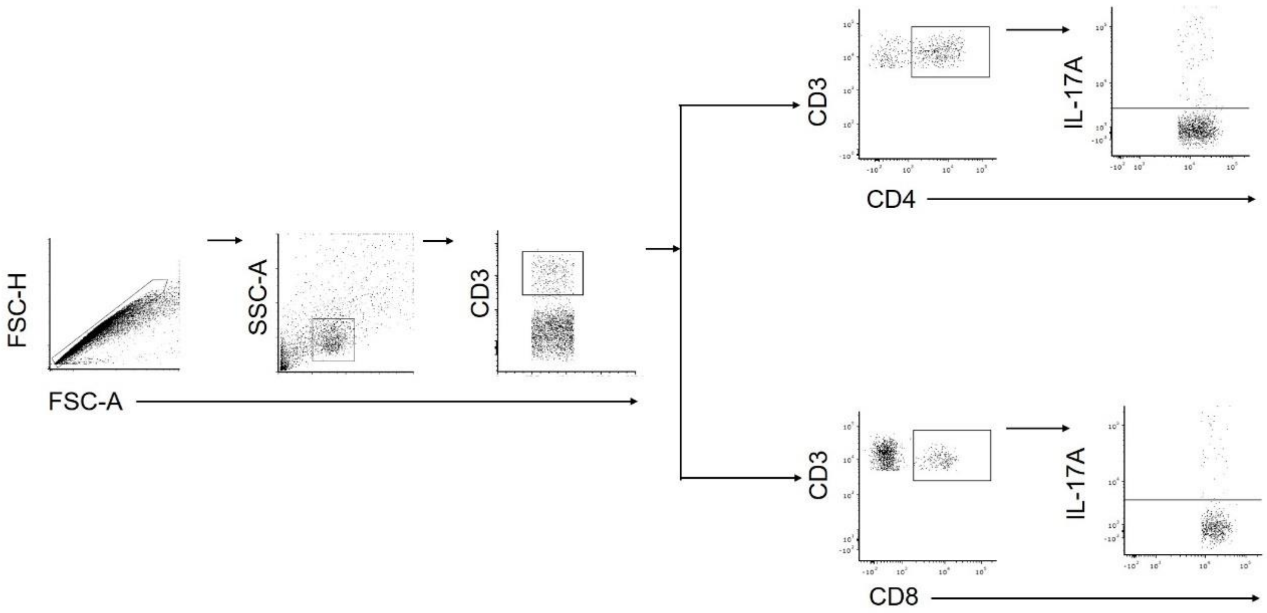

Supplementary Figure S1. Gating strategy for flow cytometric analysis. Lung cells isolated from the infant mice treated with amoxicillin were stimulated with UV-killed *S. pneumoniae* TIGR4, and stained with antibodies specific for CD3, CD4, CD8, IL-17A markers, and analyzed by flow cytometry. The cells were first plotted against FSC-H and FSCA to exclude the doublet cells and then plotted against SSC-A and FSC-A. Analysis was performed on gated CD3+ (T cells) lung cells based on a gate that mainly contains lymphocytes in the SSC-A versus FSC-A plot. CD3+ cells were displayed into CD4+ and CD8+ T cells, which were further shown to express IL-17A. Representative flow cytometric dot plot images are given.

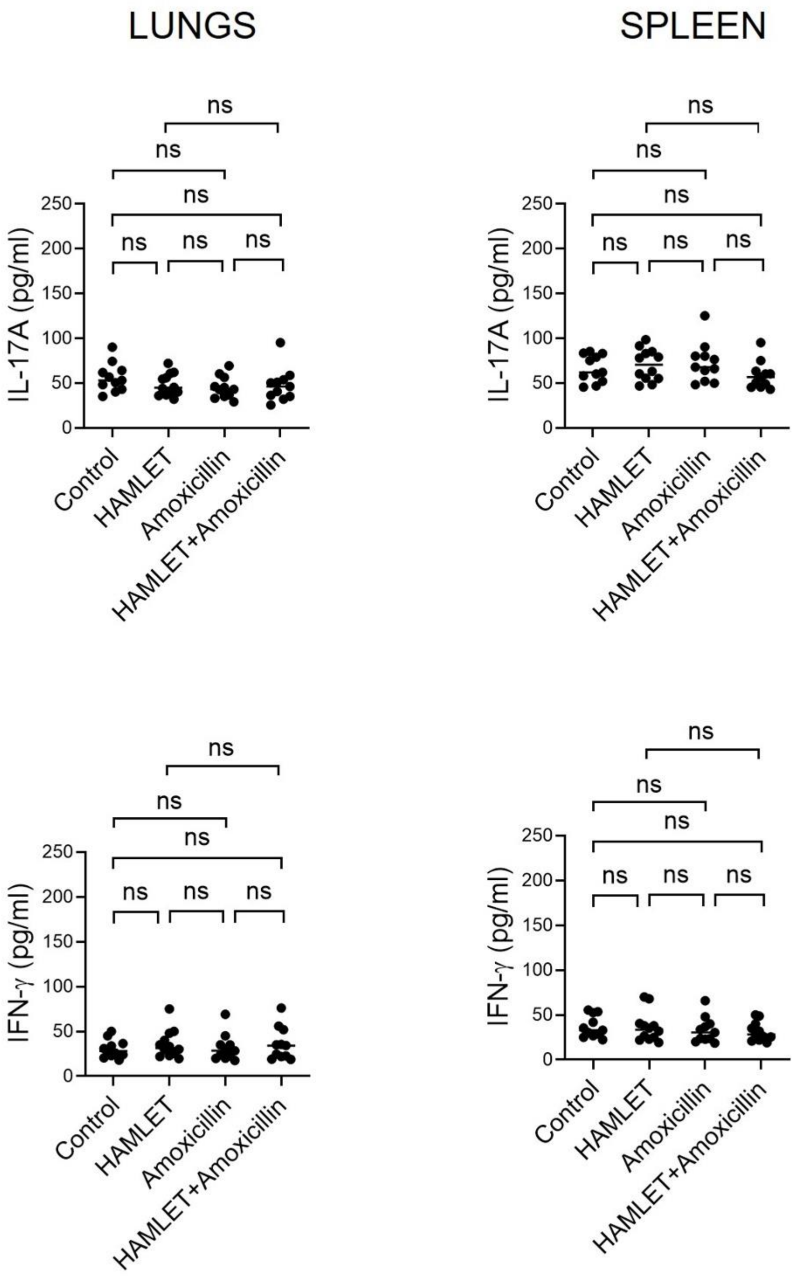

Supplementary Figure S2. IL-17A and IFN-γ production by lung cells and splenocytes without pneumococcal stimulation *in vitro*. Lung cells and splenocytes from the infant mice treated with amoxicillin, HAMLET, amoxicillin plus HAMLET or PBS (control) were cultured for 72 hours without stimulation with the UV-killed *Streptococcus pneumoniae*, and Th17 (IL-17A) and Th1 (IFN-γ) cytokine levels in the culture supernatants were measured by ELISA. Each experimental group had 11-12 mice. The data represent the mean ± SD of two independent experiments. The dots represent the data for each mouse, and the horizontal bars are the mean values for the groups. ns = non-significant. One-way ANOVA and Tukey post hoc test.

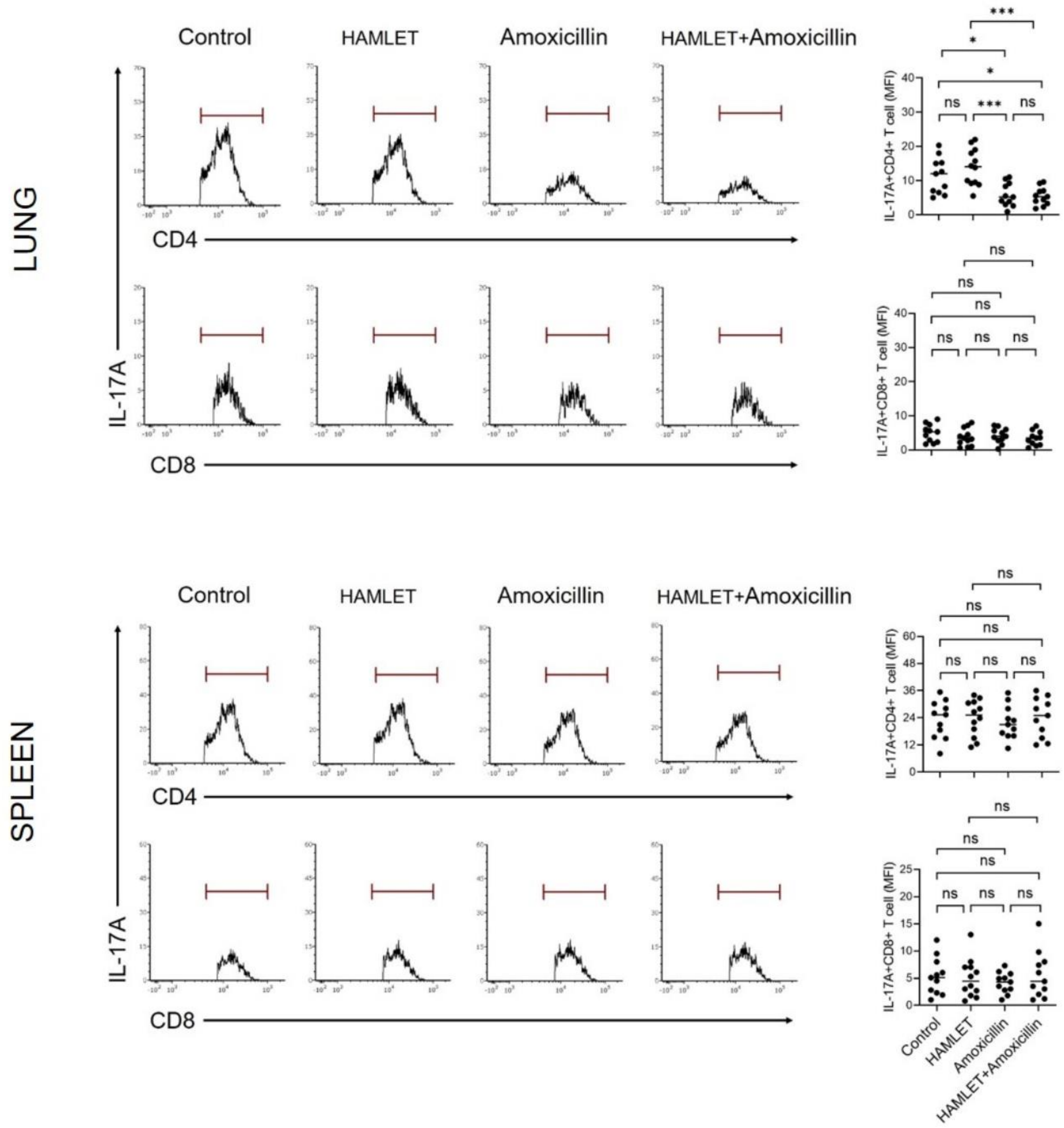

Supplementary Figure S3. Production of IL-17A by CD4+ and CD8+ T cell subsets following stimulation with killed *S. pneumoniae*. The production of IL-17A by CD4+ and CD8+ T cells in the lungs and spleen was examined using flow cytometric intracellular cytokine analysis. Images of flow cytometric histograms (left) and a summary of the mean fluorescence intensity (MFI) of IL-17A expression in CD4+ and CD8+ T cells. CD3+ cells were gated and presented as CD3+CD4+ and CD3+CD8+ T cells as described in Supplementary Figure 1. There were 11–12 infant mice in each experimental group. The information on the right graph is presented as the mean ± SD and is a summary of the two separate experiments. The horizontal bars show the mean values for the groups, and the dots show data from each individual mouse. **P*<0.05; ****P*<0.001. ns = non-significant. One-way ANOVA and Tukey post hoc test.
